# A Rapid Detection Method for H3 Avian Influenza Viruses Based on RT–RAA

**DOI:** 10.3390/ani14172601

**Published:** 2024-09-06

**Authors:** Jiaqi Li, Huan Cui, Yuxin Zhang, Xuejing Wang, Huage Liu, Yingli Mu, Hongwei Wang, Xiaolong Chen, Tongchao Dong, Cheng Zhang, Ligong Chen

**Affiliations:** 1 College of Veterinary Medicine, Hebei Agricultural University, Baoding 071000, Chinamuyingli07@163.com (Y.M.); dtch2023@163.com (T.D.); 2 Hebei Key Laboratory of Analysis and Control of Zoonotic Pathogenic Microorganism, College of Veterinary Medicine, Hebei Agricultural University, Baoding 071000, China; 3 The Animal Husbandry and Veterinary Institute of Hebei, Baoding, 071001, China; 13730474185@163.com (X.W.);

**Keywords:** H3, AIV, RT–RAA, visualization, rapid detection

## Abstract

**Simple Summary:**

The continuous evolution of the H3 subtype of avian influenza virus (AIV) poses a constant threat to the poultry industry and human health. This study successfully established a highly specific and rapid detection method for H3 AIV using real-time reverse transcription recombinase-aided isothermal amplification (RT–RAA). This method achieves accurate detection of H3 AIV by designing specific primers and probes without cross-reactivity with other viruses. The results show that the method exhibits high sensitivity and specificity and is highly consistent with the results of real-time quantitative PCR (RT–qPCR). In addition, RT–RAA amplification products can be visually observed by a portable blue light instrument, which is suitable for rapid detection in resource-constrained settings.

**Abstract:**

The continued evolution of H3 subtype avian influenza virus (AIV)—which crosses the interspecific barrier to infect humans—and the potential risk of genetic recombination with other subtypes pose serious threats to the poultry industry and human health. Therefore, rapid and accurate detection of H3 virus is highly important for preventing its spread. In this study, a method based on real-time reverse transcription recombinase-aided isothermal amplification (RT–RAA) was successfully developed for the rapid detection of H3 AIV. Specific primers and probes were designed to target the hemagglutinin (HA) gene of H3 AIV, ensuring highly specific detection of H3 AIV without cross-reactivity with other important avian respiratory viruses. The results showed that the detection limit of the RT–RAA fluorescence reading method was 224 copies/response within the 95% confidence interval, while the detection limit of the RT–RAA visualization method was 1527 copies/response within the same confidence interval. In addition, 68 clinical samples were examined and the results were compared with those of real-time quantitative PCR (RT–qPCR). The results showed that the real-time fluorescence RT–RAA and RT–qPCR results were completely consistent, and the kappa value reached 1, indicating excellent correlation. For visual detection, the sensitivity was 91.43%, the specificity was 100%, and the kappa value was 0.91, which also indicated good correlation. In addition, the amplified products of RT–RAA can be visualized with a portable blue light instrument, which enables rapid detection of H3 AIV even in resource-constrained environments. The H3 AIV RT-RAA rapid detection method established in this study can meet the requirements of basic laboratories and provide a valuable reference for the early diagnosis of H3 AIV.

## 1. Introduction

Avian influenza virus (AIV) is a genus of *Alphainfluenzavirus* and is considered one of the most serious zoonotic diseases. AIV poses a significant threat to the global poultry industry and can have a profound impact on human life and health [1]. Its genome structure consists of eight single-stranded negative RNA segments, which together encode at least 10 core viral proteins [2]. AIVs can be classified into 18 HA subtypes and 11 NA subtypes based on the antigenicity of their surface proteins, hemagglutinin (HA) and neuraminidase (NA) [3,4]. AIV has a wide host range and usually exists naturally in poultry and wild birds, with wild waterfowl being the main reservoirs of most type A influenza viruses. Additionally, AIV can potentially cross species barriers to infect mammals, including humans [5]. Among them, H3 AIV is relatively low in pathogenicity and usually causes mild or no obvious symptoms in birds, making it widespread in birds, humans, and other mammals [6]. H3NX AIV has various subtypes, with H3N2 and H3N8 being the main subtypes. However, due to frequent mutation and recombination, the pathogen ecology of avian influenza virus has become more complex. This phenomenon is not only closely related to changes in the virulence and transmission mode of the virus but also profoundly affects the adaptability of the virus to mammals, thus posing a potentially serious threat to both animal and human health [7]. In 2022, a novel strain of Alphainfluenzavirus influenzae H3N8 virus was detected in Henan Province, China, causing infection in a 4-year-old boy, marking the first human infection with this strain [8]. In the same year, a 5-year-old boy was also found to be positive for H3N8 AIV in Changsha, Hunan Province. After in-depth exploration and tracing by researchers, it was discovered that the new H3N8 virus originated in chickens. The avian influenza H3N8 virus strain is a novel virus produced by dynamic gene reassortment. The strain acquired the H3 gene from the Eurasian lineage virus, the N8 gene from the North American lineage virus, and the internal genes from the G57 genotype H9N2 virus [9]. H3N8 AIVs continue to evolve and mutate, further increasing the risk of virus transmission to infect humans [10]. In addition, studies have shown that H3NX AIV can recombine gene fragments with other influenza virus subtypes, potentially giving rise to new strains and triggering influenza outbreaks [7,11]. The prevalence of the H3 subtype avian influenza virus (AIV) in the Chinese poultry industry varies across different regions and types of poultry. Recent surveillance data indicate that H3 AIVs are frequently detected in live poultry markets and farms. A study revealed that H3 AIVs have been isolated from various poultry environments, including live poultry markets, farms, and slaughterhouses across China. The data suggest a significant presence of H3 AIVs in these environments, highlighting the virus’s wide geographic spread and diversity [12]. In a study focusing on live poultry markets, H3 AIVs were found in 5.2% of chicken samples, 8.8% of duck samples, and 6.3% of pigeon samples, while no H3 AIVs were detected in geese samples [13].

Several detection methods for H3 subtype avian influenza viruses have been established. However, these methods still need to be improved in terms of operability, sensitivity, and field applicability. Virus isolation and identification are regarded as the “gold standard” for detecting avian influenza virus, but their detection times are long and they are not suitable for rapid large-scale detection [14]. The immunological detection technique is relatively simple and suitable for the preliminary screening of a large number of samples but it has the problem of false positives, which affects the specificity of detection [15]. Quantitative real-time PCR (RT–qPCR) is a molecular biological detection method that, although fast, relies on expensive detection equipment and specialized laboratory conditions [16]. Loop-mediated isothermal amplification (LAMP) has become a popular diagnostic method, but designing specific primers for the target sequence is complicated and difficult [17]. To improve the detection of H3 subtype avian influenza viruses, this study established a rapid detection method utilizing reverse transcription recombinase-aided amplification (RT–RAA). RT–RAA can amplify DNA/RNA in a relatively short time (5–30 min) and in isothermal conditions (37–42 °C) [18]. This technique uses specific primers and probes as well as an efficient recombinant enzyme system to accurately detect the target nucleic acid sequence [19]. The RT-RAA method is based on the principle of reverse transcription and recombinase-aided amplification. Briefly, the target RNA is first reverse transcribed into cDNA using a reverse transcriptase enzyme. Then, the cDNA is amplified using a specific primer set and an enzyme system that includes a recombinase, a single-stranded DNA binding protein, and a thermostable DNA polymerase. The probe used in this method is designed to specifically bind to the amplified cDNA, allowing for the detection of the target RNA. The probe is typically labeled with a fluorescent dye, which enables visual detection of the amplification product. The enzyme system used in RT-RAA is optimized to provide high amplification efficiency and specificity, making it suitable for the detection of low levels of target RNA in complex samples. It is suitable not only for DNA amplification but also for RNA detection, and can simultaneously detect multiple pathogens [20]. In addition, RT–RAA can be combined with other technologies to achieve rapid visual detection, further simplifying the operation process and improving the detection efficiency [21]. In addition, the equipment and reagents used in RT–RAA are relatively simple and inexpensive, which further improves its practical application value [18]. RT-RAA is an isothermal amplification technique that can detect viral RNA without the need for thermal cycling, which is required in traditional PCR methods. This allows for rapid detection of H3 AIV within a short time frame, typically within 30 min to an hour, depending on the optimized conditions. Rapid detection is crucial for early disease intervention and control, especially in the case of highly contagious viruses such as avian influenza [22]. RT-RAA has been shown to possess high sensitivity and specificity for detecting viral RNA [23]. This is achieved through the use of specific primers and probes designed to target conserved regions of the viral genome. In the case of H3 AIV, the technique can detect low viral loads, making it suitable for early-stage infections where viral titers may be low. RT-RAA does not require sophisticated laboratory equipment or highly trained personnel. The isothermal nature of the reaction allows for the use of portable devices, making it feasible for field-based testing. This portability is particularly important in remote areas or during outbreaks where access to laboratory facilities may be limited. Compared to traditional PCR methods, RT-RAA is generally more cost-effective due to its simplicity and the reduced need for specialized equipment. This makes it a more viable option for large-scale screening and surveillance programs, particularly in developing countries. The combination of speed, sensitivity, specificity, and portability makes RT-RAA an ideal candidate for developing point-of-care testing (POCT) devices for H3 AIV. POCT devices can provide rapid results at the point of care, enabling prompt clinical decision-making and improved outcomes. Rapid and accurate detection of H3 AIV is crucial for epidemiological surveillance. By identifying infected birds early, control measures can be promptly implemented to prevent the spread of the virus, thereby minimizing economic losses to the poultry industry and protecting public health. Measures that can be taken include isolating and treating infected birds, thoroughly disinfecting poultry houses and their surrounding environments, promoting and implementing vaccination programs, enhancing monitoring of bird health status and regular virus testing, controlling and reducing personnel and material movement between farms, and raising awareness of epidemic prevention among farmers and related personnel while providing them with training. Through these comprehensive measures, the spread of H3 subtype avian influenza can be effectively contained, safeguarding both the poultry industry and public health. In summary, the development of RT-RAA for the detection of H3 AIV represents a significant advancement in viral diagnostics. Its rapid, sensitive, specific, and cost-effective nature, combined with its potential for portability and POCT, make it an invaluable tool for early disease detection, control, and epidemiological surveillance.

Existing studies have shown that two major subtypes of H3NX influenza viruses, H3N2 and H3N8, can cross the species barrier and pose a serious threat to animal and human health. Therefore, it is necessary to establish a detection method for H3 subtype avian influenza virus [24] In our study, specific primers and probes targeting the HA gene of H3 AIV were designed, enabling us to develop a rapid detection method based on visualized real-time fluorescence RT–RAA technology. This method can aid in the diagnosis of H3 AIV, aiding in the prompt and effective prevention and timely and effective control of the disease to ensure the safety of the livestock and poultry industry as well as human health.

## 2. Materials and Methods

### 2.1. Source of Viruses and Clinical Samples

Two virus strains stored in the laboratory were used in the study: A/chicken/Hebei/ZC55/2023 (H3N2) and A/chicken/Hebei CY59/2023 (H3N8). In 2024, we collected 68 clinical samples (including tracheal, lung, oropharyngeal, and anal swabs) with suspected AIV infection from different parts of Hebei Province.

### 2.2. Design of RT–RAA Primer and Probe Design

H3 AIV-HA gene sequences (GenBank No. KT022317.1 and OP024225.1) were downloaded from the GenBank database and analyzed using DNASTAR software (version 7.0). SnapGene software (version 4.3.6) was employed to design primers and probes. The optimal primers and probes were screened according to our previous study [25]. Specifically, it is necessary to screen the best reverse primers through the use of randomly selected forward primers and then use these selected reverse primers to identify good forward primers. Through two rounds of screening, the aim was to identify more sensitive primer pairs and finally determine the best pair of primers. The positions and sequences of the primers and probes are shown in Table 1. The primers and probes used in this study were synthesized by Sangon Biotech Co., Ltd., (Shanghai, China).

### 2.3. Nucleic Acid Extraction

According to the instructions of the QIAamp Viral RNA Mini Kit (Hilden, Germany), the RNA of the following strains and viruses in the samples were extracted: A/swine/Hebei/SD17/2023 (H1N1), A/chicken/Hebei/ZC55/2023 (H3N2), A/chicken/Hebei/CY59/2023 (H3N8), A/chicken/Hebei/HB777/2006 (H5N1), A/chicken/Hebei/CK05/2019(H5N6), A/quail/Hebei/CH06-07/2018(H7N9), A/chicken/Hebei/015/2019(H9N2), infectious laryngotracheitis virus (ILTV), infectious bronchitis virus (IBV), Newcastle disease virus (NDV), and 68 clinical samples of suspected AIV infection. RNase-free water was used as the negative control, and the negative control samples underwent the same processing steps as the experimental samples. All the extracted RNA was stored at −80 °C for no more than 24 h after extraction.

### 2.4. RT–RAA Amplification

The RNA was amplified using an RNA constant temperature rapid amplification kit (Anpu Future Biotechnology Co., Ltd., Weifang, China). According to the instructions, a 25 μL reaction mixture consisting of 14.7 μL of Buffer A, 4.75 μL of RNase-free water, 1.0 μL of forward primer (initial concentration was 10 μM), 1.0 μL of reverse primer (initial concentration was 10 μM), 0.3 μL of probe (initial concentration was 10 μM), 2.0 μL of nucleic acid template, and 1.25 μL of Buffer B was prepared. Subsequently, amplification was performed on a Gentier 96E qPCR machine (Tianlong, Xian, China) and the fluorescence signal was monitored (42 °C, 30 min, 1 amplification cycle/min). In the negative control, the fluorescence signal was stable during the amplification period of RT-RAA and formed the baseline. Fluorescence thresholds were set based on negative control findings to determine the initiation of amplification. When the fluorescence signal of the sample is lower than the baseline and there is no amplification curve, it is considered negative. It is considered positive when it is above baseline and an amplification curve is present. In addition, the RT–RAA amplification products were visualized using a portable blue-ray imager with an excitation wavelength of 480 nm (TIANGEN, Beijing, China).

### 2.5. RT–qPCR Assay

The RT–qPCR kit was purchased from Novizan Biotechnology (Q223, Nanjing, China) and amplified according to the instructions using primers and probes reported in previous studies [26]. The 25 μL reaction mixture consisted of 12.5 μL of 2× one step U+ Mix, 1.5 μL of one step U+ Enzyme Mix, 1.0 μL of forward primer (10 μM), 1.0 μL of reverse primer (10 μM), 0.5 μL of probe (10 μM), 2.0 μL of nucleic acid template, and 6.5 μL of RNase-free water. The reaction tube was placed in a Gentier 96E qPCR machine (Tianlong, Xian, China) and the fluorescence signal was monitored. The procedure was set as follows: 55 °C for 15 min, 95 °C for 30 s, followed by 40 cycles at 95 °C for 10 s, and 60 °C for 30 s.

### 2.6. Specific Analysis

The following RNAs were used as templates for real-time fluorescent RT–RAA amplification experiments to verify the specificity of this method: H1N1, H3N2, H3N8, H5N1, H5N6, H7N9, H9N2, ILTV, IBV, and NDV. These experiments were conducted using prescreened specific primers and probes.

### 2.7. Sensitivity Analysis

A laboratory-preserved H3 IAV-HA plasmid (pMD18-T-HA) was used for sensitivity analysis. The H3 AIV-HA plasmid was diluted in a tenfold series to concentrations ranging from 10^5^ to 10^0^ copies/2 µL. An amount of 2 µL of each plasmid dilution was used as a template to evaluate the sensitivity of real-time RT–RAA. For comparison, parallel detection was performed on the same template using the RT–qPCR method. To more accurately analyze the detection limit, eight independent runs were performed in both analyses, and the data were analyzed using probit regression in Statistical Product and Service Solutions (SPSS) software (version 19.0).

### 2.8. Repeatability and Stability Analysis

To evaluate the intragroup and intergroup reproducibility of the RT–RAA method, high, medium, and low concentrations of H3 AIV-HA plasmids (10^7^, 10^5^, and 10^3^ copies per reaction) were used for repeated detection in a single run or three independent runs at different times, and the coefficient of variation (CV) of the threshold time was calculated.

### 2.9. Clinical Sample Testing

The real-time RT–RAA method was used to detect 68 clinical samples. For comparison, RT–qPCR was used for parallel detection of the same samples and the coincidence rates of the two methods were compared.

### 2.10. Statistical Analysis

Probit regression analyses at a 95% coincidence level were applied in SPSS software to determine the limit of amplification. Kappa statistics were applied to compare the coincidence rate between real-time RT–RAA and RT–qPCR detection.

## 3. Results

### 3.1. Optimal Primer Screening for RT–RAA

**Figure 1:**
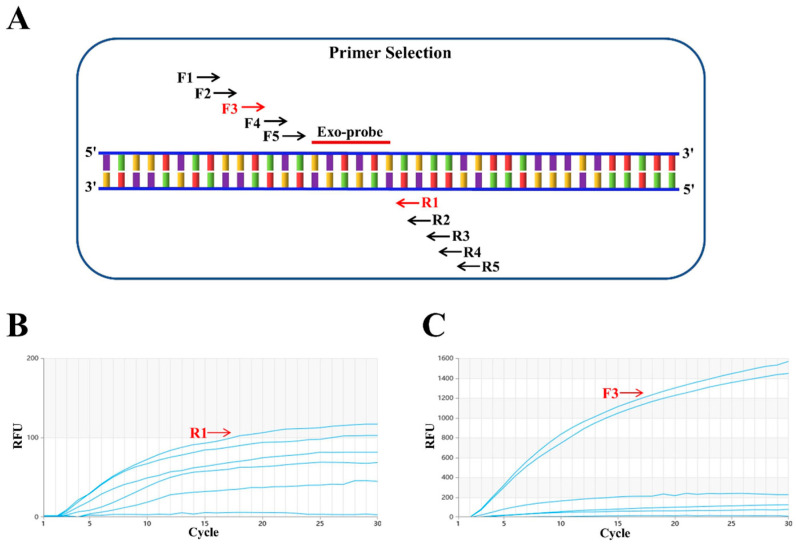
Primer screening. (**A**) Schematic diagram of primer screening for RT–RAA detection. (**B**) Downstream primer screening results. The forward primer F1 was randomly selected to screen all five reverse primers, with R1 performing the best. (**C**) Upstream primer screening results. The selected reverse primer R1 was used to screen five forward primers, with F3 performing the best.

**Figure 2:**
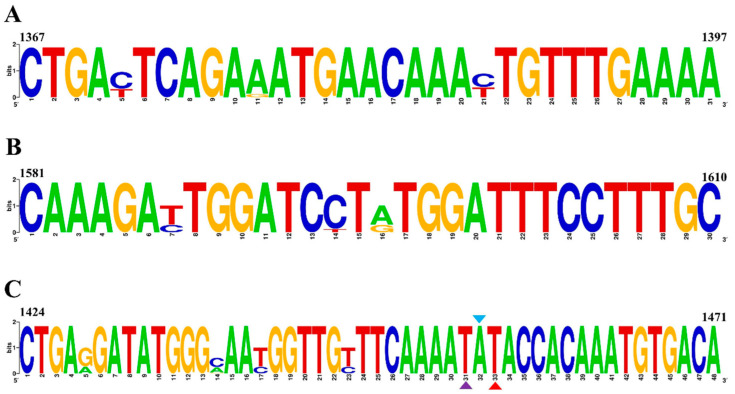
Primers and probes for RT–RAA. (**A**) Upstream primer (F1367–1397), (**B**) downstream primer (R1581–1610), (**C**) probe (p1424–1471). The purple triangle indicates the fluorophore-labeled residue (FAM), the red triangle indicates the quencher-labeled residue (BHQ1), and the blue triangle represents the THF: tetrahydrofuran spacer. Green for adenine (A), red for thymine (T), orange for guanine (G) and blue for cytosine (C).

First, an Exo probe named p1424–1471 was designed (see Figure 1A), and the details are provided in Figure 2C. Next, based on p1424–1471, five upstream candidate primers (F1, F2, F3, F4, F5, shown in Figure 1A) and five downstream candidate primers (R1, R2, R3, R4, R5, also see Figure 1A) were designed. Specifically, the upstream primer F1 was randomly selected and paired with five downstream primers (R1, R2, R3, R4, R5) for screening. The experimental results demonstrated that the amplification effect was most pronounced when the products were paired with R1 (Figure 1B). Subsequently, R1 was tested with five upstream primers (F1, F2, F3, F4, F5), revealing that the amplification effect was optimal when R1 was paired with F3 (see Figure 1C). Therefore, among all primer pairs, the combination of F3 (Figure 2A) and R1 (Figure 2B) exhibited superior performance. Finally, the primer pair F3/R1 and the probe p1424–1471 were selected for real-time RT–RAA detection.

### 3.2. Specific Analysis

**Figure 3:**
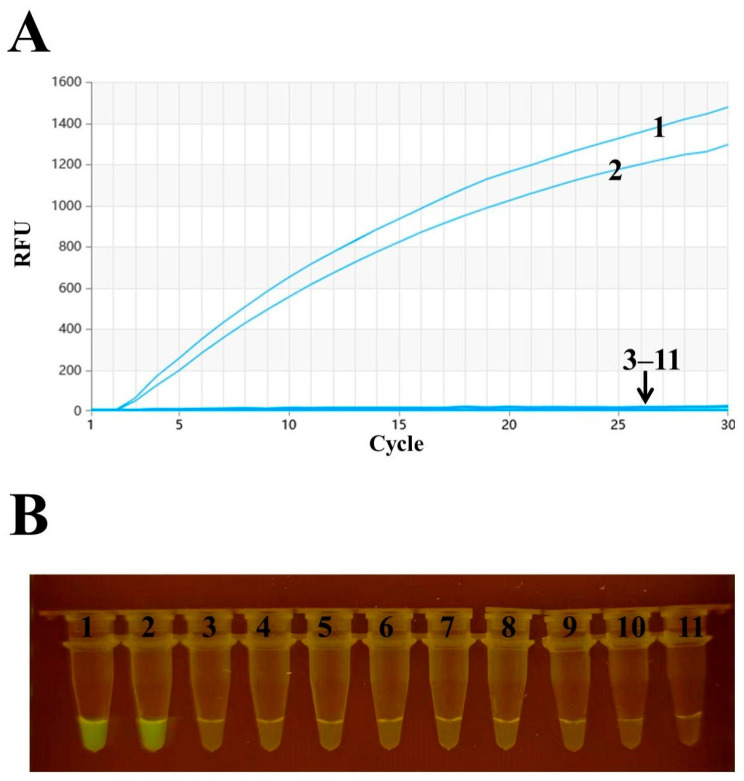
H3 AIV specificity detection. (**A**) Real-time fluorescence detection results of RT–RAA. (**B**) RT–RAA detection results using a portable blue light instrument. Numbers 1–11 represent H3N2, H3N8, H1N1, H5N1, H5N6, H7N9, H9N2, ILTV, IBV, NDV, and the negative control, respectively.

To conduct an in-depth specificity analysis, this study not only extracted RNA from H3N2 AIV and H3N8 AIV but also included nucleic acids from various other viruses, specifically H1N1, H5N1, H5N6, H7N9, H9N2, ILTV, IBV, and NDV. Subsequently, the RT–RAA method was used to detect all the extracted viral nucleic acids. The experimental results are shown in Figure 3, in which only the H3N2 and H3N8 virus samples showed positive reactions, while the H1N1, H5N1, H5N6, H7N9, H9N2, ILTV, IBV, NDV, and RNase-free water control groups all showed negative results. In particular, the amplified products of the RT–RAA experiment can be directly observed with the naked eye under a portable blue light instrument (with an excitation wavelength of 480 nm) (see Figure 3B). The above experimental results demonstrated that the real-time RT–RAA detection method has good specificity for H3 AIV.

### 3.3. Sensitivity Analysis

**Figure 4:**
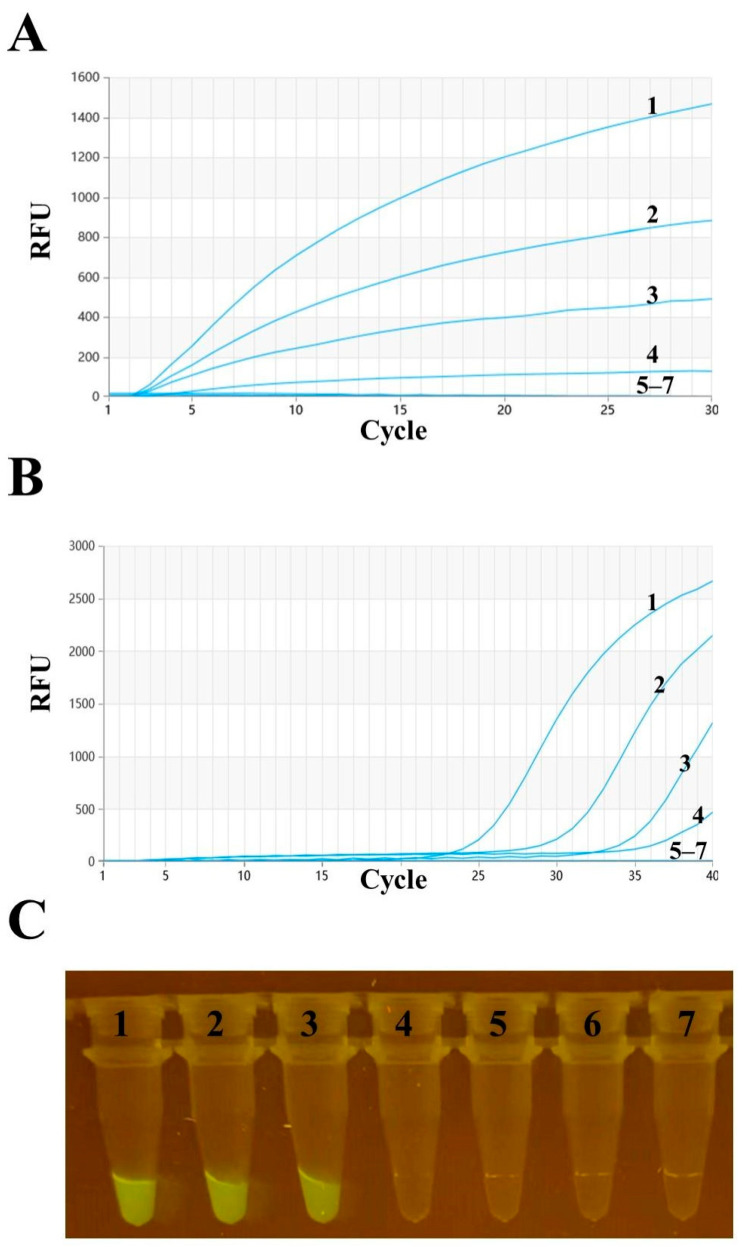
Sensitivity detection of H3 AIV. Numbers 1–7 represent 10^5^ to 10^0^ copies per reaction and a negative control, respectively. (**A**) Sensitivity of H3 AIV detection by real-time fluorescence reading via RT–RAA. (**B**) Sensitivity of H3 AIV detection by RT–qPCR. (**C**) Sensitivity of H3 AIV detection by visual RT–RAA.

A sensitivity analysis was conducted using RT–RAA and RT–qPCR methods to detect templates made from serially diluted H3 AIV-HA plasmids (10^5^ to 10^0^ copies). Based on the real-time fluorescence readings from both detection methods, the detection limit for each reaction was 10^2^ copies per reaction (Figure 4A,B), with a visualization result of 10^3^ copies per reaction (Figure 4C). Further probit regression analysis indicated that the detection limit at a 95% confidence interval was 224 copies per reaction for the RT–RAA method, 171 copies per reaction for the RT–qPCR method, and 1527 copies per reaction for the visualization result of RT–RAA.

### 3.4. Repeatability and Stability Analysis

To analyze the repeatability and stability, the RT–RAA method was used to detect three concentrations of H3 AIV-HA plasmids: 10^7^ copies/reaction, 10^5^ copies/reaction, and 10^3^ copies/reaction. These tests were repeated three times, and the results are shown in Table 2. The intra-assay CVs were 4.22%, 4.64%, and 5.75% for the three concentrations, respectively, while the inter-assay CVs were 5.54%, 6.23%, and 6.37%, respectively. Both the intra-assay and inter-assay CVs were less than 6% and 7%, respectively (Table 2), indicating that the RT-RAA method has excellent repeatability and stability.

### 3.5. Clinical Sample Testing

To evaluate the performance of the RT–RAA method for detecting H3 AIV, 68 clinical tissue samples were tested using RT–RAA and the results were compared with those obtained using RT–qPCR. The positive results indicate nucleic acid of H3 AIV was detected in the samples, while the negative results suggest that nucleic acid of the virus was not detected. As shown in Table 3, among the 68 samples, 35 tested positive for RT-RAA. The RT-qPCR-positive results were consistent with RT–RAA. The sensitivity of real-time RT–RAA detection was 100% (35/35), the specificity was 100% (33/33), and the accuracy was 100% (68/68). The kappa value for the two analyses was 1, indicating a good correlation. Additionally, among the 35 positive samples detected by RT–RAA, 32 were positive by visual detection. The sensitivity of visual detection was 91.43% (32/35), the specificity was 100% (33/33), and the accuracy was 95.59% (65/68). The kappa value was 0.91, indicating good correlation. This fully demonstrates that the RT–RAA method established in this study has good detection performance and is feasible for on-site detection.

## 4. Discussion

H3 AIV is a low pathogenicity avian influenza virus subtype commonly found in poultry. After animals are infected with the virus, they often show no obvious symptoms or only mild clinical symptoms. Therefore, the potential impacts on farming and human health are often overlooked. However, this neglect has provided opportunities for the continued spread and evolution of H3 AIV in nature, which in turn has caused considerable economic losses to the poultry industry [24]. Notably, H3 AIV is not limited to infecting poultry; it may change its pathogenicity through mutation or recombination and then cross the interspecific barrier to infect humans [27]. Studies have shown that some H3N8 viruses can bind to both avian and human receptors and can be transmitted in guinea pigs through respiratory droplets, suggesting a certain potential pandemic [28]. The WHO was notified by China in April 2022 of the first-ever recorded human infection with the H3N8 subtype of AIV [29]. H3N8 AIV has shown low pathogenicity in poultry and has not been considered a priority for control. However, this neglect has provided opportunities for the continued spread of the virus [28]. The continuous evolution of H3 AIV and the emergence of H3N8 subtype avian influenza virus infection highlight its threat to public health and human life and health [30]. Therefore, establishing a rapid on-site diagnostic method for H3 AIV is particularly important for reducing virus transmission, minimizing economic losses, and conducting long-term monitoring of H3 AIV.

At present, various detection methods for H3 AIV have been developed, such as RT–PCR, RT–qPCR, and LAMP. However, these methods have several limitations. Although the traditional RT–PCR method is widely used, it has a high detection limit and relies on agarose gel electrophoresis to read the results, which increases the complexity of the operation [31]. The RT–qPCR method, while enhancing sensitivity, demands costly thermal cycling equipment and skilled technicians, which can be challenging in resource-constrained settings [32]. On the other hand, the LAMP method involves a more intricate detection process since it necessitates the design of at least two pairs of primers, increasing the difficulty and expense of the experiment [33]. Considering the restrictions of current tests and the potential menace of H3 AIV to public health and the poultry industry, there is a pressing need for the development of a test capable of swift, precise, and sensitive detection of H3 AIV, facilitating prompt control measures to minimize virus transmission risks.

Our previous study established an RT–RAA rapid detection method for *Alphainfluenzavirus* [34], which provides a rapid, sensitive and reliable detection tool for *Alphainfluenzavirus*. However, there is no RT–RAA method for H3 AIV detection that allows direct visualization. In this study, we established a novel visual real-time fluorescent RT–RAA method for detecting H3 AIVs by designing specific primers and probes targeting the HA gene of H3 AIVs. We aligned gene sequences from the GenBank database to ensure the optimal performance of our detection method. Real-time RT–RAA and RT–qPCR detection showed a limit of detection (LOD) of 100 copies/reaction for H3 AIV per reaction. Probit regression analysis indicated that the LOD at the 95% confidence interval was 224 copies/reaction for RT–RAA and 171 copies/reaction for RT–qPCR. Interestingly, the amplified products of real-time RAA could be visualized under a portable blue light instrument with an LOD of 1000 copies/reaction. Similarly, probit regression analysis revealed that the LOD for visual RT–RAA detection at a 95% confidence interval was 1527 copies/reaction, making on-site detection feasible. Additionally, this method demonstrated excellent specificity for H3 AIV, showing no cross-reactivity with viruses such as H1N1, H5N1, H5N6, H7N9, H9N2, ILTV, IBV, and NDV. Among the 68 samples, 35 tested positive according to RT–RAA and all 35 RT–RAA-positive samples were also positive according to RT–qPCR. The sensitivity, specificity, and accuracy of real-time RT–RAA detection were 100% (35/35), 100% (33/33), and 100% (68/68), respectively. Both analyses showed a kappa value of 1, indicating good correlation. Furthermore, among the 35 RT–RAA-positive samples, 33 were positive by visual detection. The sensitivity, specificity, and accuracy of visual detection were 91.43% (32/35), 100% (33/33), and 95.59% (65/68), respectively. The kappa value was 0.91, indicating good correlation. This fully demonstrates that our established RT–RAA method exhibits excellent detection performance and is feasible for on-site testing. Researchers have developed an RT–LAMP method for detecting H3 AIV with an LOD of 1000 copies/reaction, which is comparable to our method in terms of sensitivity [35].

Additionally, the RT–LAMP method requires the design of three pairs of primers, making it relatively complex. Our RT–RAA method requires only one pair of primers, and the amplified products can be directly visualized, enhancing the feasibility of on-site detection. Moreover, an RT–qPCR method for detecting H3 AIV has also been established [26], with an LOD of 210 copies/reaction, which is comparable to the sensitivity of our method. The detection time for RT–qPCR is approximately 120 min, whereas RT–RAA requires only 30 min, making RT–RAA significantly faster and highly valuable in scenarios where rapid detection results are needed. Compared to RT–qPCR technology, RT–RAA detection offers several notable advantages. First, the amplified products of RT–RAA detection have special visualization characteristics. With the assistance of a portable blue light imaging device with an excitation wavelength of 480 nm, these amplified products can be directly observed by the naked eye. This feature simplifies the detection process and enhances its intuitiveness and convenience. Second, the experimental conditions for RT–RAA are relatively simple and flexible. The experiment can be conducted in a constant temperature environment ranging from 37–42 °C, which can be achieved through simple methods such as water baths, metal baths, or even body temperature [36]. This convenient temperature requirement makes RT–RAA particularly suitable for on-site operations or use in resource-limited environments, greatly enhancing its practicality and range of applications. Therefore, it is entirely feasible to develop RT–RAA technology into a kit suitable for rapid on-site detection, providing significant convenience for field testing.

## 5. Conclusions

This study successfully developed a visual RT-RAA detection approach aimed at the H3 AIV-HA gene, enabling efficient identification of H3 AIV. This method shows remarkable rapidity, simplicity, and reliability, indicating that it has broad application prospects in the field of H3 AIV detection. Furthermore, this study realized visual observation of RT–RAA amplification products with a portable blue light instrument. This innovative feature greatly improves the field application and real-time detection capabilities of the method, enabling swift identification of H3 AIV in a sample without the need for complex laboratory equipment or professional assistance. This visual RT–RAA detection method is particularly valuable in resource-constrained situations or emergencies. It can not only quickly identify virus infection and provide key intelligence for the timely implementation of prevention and control measures but also serve as an important early warning function in the initial stage of the epidemic, which has far-reaching significance for enhancing public health security and the strength of avian influenza prevention and control. Despite the promising results, this study has several limitations. Firstly, while effective for H3 AIV, the RT-RAA method has not been extensively tested across a wide range of environmental conditions or in various field settings, which could affect its robustness and reliability in real-world applications. Secondly, the method’s performance needs further validation with a larger and more diverse sample size to ensure its generalizability and accuracy across different strains and populations of H3 AIV. Thirdly, while the visual detection component of RT-RAA enhances its utility for on-site testing, it may still require some level of technical expertise to interpret results accurately, potentially limiting its use in very resource-limited or untrained settings. Fourthly, although as of now, the H3N8 subtype avian influenza virus has infected three human cases worldwide, the risk of this virus spreading among humans is considered low. However, due to the continuous evolution of influenza viruses, global monitoring to detect virological, epidemiological, and clinical changes that may affect human or animal health still needs to be strengthened. Lastly, the study did not compare the cost-effectiveness of RT-RAA with other existing methods, which is a critical factor for widespread adoption, particularly in low-resource environments.

## Figures and Tables

**Table 1:** The primers and probes used in H3 AIV real-time RT–RAA assays.

Primers/Probe	Sequences (5’→3’)	Position
H3-F1	CAAAAGTGGATCTATGGTCTTATAATGCAG	1295–1324
H3-F2	GGTCTTATAATGCAGAGCTTCTTGTTGCTC	1310–1339
H3-F3	CTGACTCAGAAATGAACAAACTGTTTGAAAA	1367–1397
H3-F4	GTTTGAAAAGACCAGAAGGCAGCTAAGAGA	1389–1418
H3-F5	AAAAGACCAGAAGGCAGCTAAGAGAGAATG	1394–1423
H3-R1	GCAAAGGAAATCCATAGGATCCAATCTTTG	1581–1610
H3-R2	CAATACAACACAAAGCAAAAAGCATGATAT	1612–1641
H3-R3	GCCCACATAATGAACCCCAACAATACAACA	1632–1661
H3-R4	CTTTCTGGCAAGCCCACATAATGAACCCCA	1643–1672
H3-R5	TGCACCTAATGTTGCCTTTCTGGCAAGCCC	1658–1687
H3-probe	CTGAGGATATGGGCAATGGTTGTTTCAAAA(FAM-dT)(THF)(BHQ1-dT)ACCACAAATGTGACA [C3-spacer]	1424–1471

**Table 2:** Results of repeatability and reproducibility detected by real-time RAA assay.

Plasmid Concentration	Repeatability (Intra-Batch Assay)			Reproducibility (Inter-Batch Assay)		
	Mean	SD	CV (%)	Mean	SD	CV (%)
High (10^7^)	95.67	4.04	4.22	102.67	5.69	5.54
Medium (10^5^)	184.00	8.54	4.64	182.33	11.37	6.23
Low (10^3^)	356.67	20.50	5.75	365.33	23.29	6.37

Mean: average threshold times (seconds) of three independent real-time RAA reactions. SD: standard deviation; CV: coefficient of variation.

**Table 3:** Comparison of H3 AIV real-time RT–RAA with RT–qPCR in clinical samples.

Assay		RT–qPCR		Sensitivity	Specificity	Kappa
		Positive	Negative			
Real-time RT–RAA (via real-time fluorescence read-out)	Positive	35	0	100%	100%	1
	Negative	0	33			
	Total (68)	35	33			
Real-time RT–RAA (via visual detection)	Positive	32	0	91.43%	100%	0.91
	Negative	3	33			
	Total (68)	35	33			

## Data Availability

The original contributions presented in this study are included in the article. For further inquiries, please contact the corresponding authors.
